# Supplementary material for: Dynamic Imaging of Glucose and Lactate Metabolism by 13C-MRS without Hyperpolarization
Source: Sci Rep. 2019 Mar 4;9:3410. doi: 10.1038/s41598-019-38981-1 (PMC6399318; doi:10.1038/s41598-019-38981-1)

**Supporting Information for:**

**Dynamic Imaging of Glucose and Lactate Metabolism by ^13^C-MRS without Hyperpolarization**

Jeffrey R. Brender^1^; Shun Kishimoto^1^; Hellmut Merkle^2^; Galen Reed,^3^ Ralph E. Hurd;^3^ Albert P. Chen;^3^ Jan Henrik Ardenkjaer-Larsen^3,4^ , Jeeva Munasinghe^2^, Keita Saito^1^; Tomohiro Seki,^1^ Nobu Oshima^1^; Kazu Yamamoto^1^; Peter L. Choyke^1^, James Mitchell^1^; ­Murali C. Krishna^1^

^1^Center for Cancer Research, NCI, National Institutes of Health ^2^NINDS, National Institutes of Health ^3^GE HealthCare ^4^Department of Electrical Engineering, Technical University of Denmark

**Supporting Information Figures**


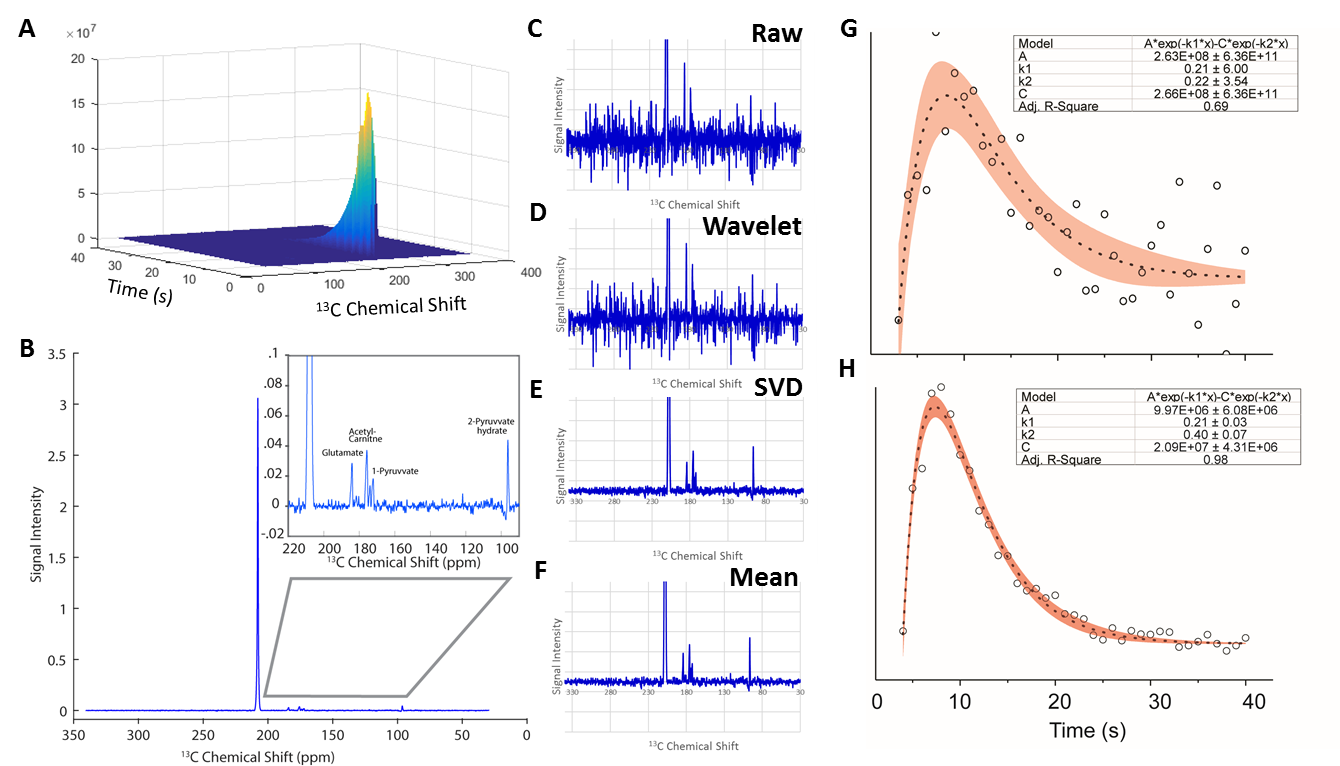


**Figure S1 SVD rank reduction improves the precision of kinetic fitting. (A)** The raw signal resulting from the brain of an Wistar rat after injection of an 37 mg bolus of pyruvate ^13^C-labeled at the 2 position. **(B)** Spectrum averaged over the 40 s time course. The signal has a very high dynamic range with the metabolites having an intensity only 0.5% of the main 2-pyruvate peak. **(C)** Spectrum 20 seconds after injection. In the raw signal, the metabolites are near the noise level, which is not significantly improved by **(D)** wavelet denoising**.** By contrast, SVD rank reduction to a rank of 5 results in a significant reduction of noise **(E)** so that the signal resembles the spectra averaged over all time points **(F)**. For glutamate at 184 ppm, the noise in the baseline of the raw signal means the curve fitting is ill-conditioned and the kinetic constants cannot be recovered with any accuracy. **(H)** Improvement in the signal to noise at longer time points by SVD removes the instability and allows reconstruction of the kinetics with greater precision.

­­­
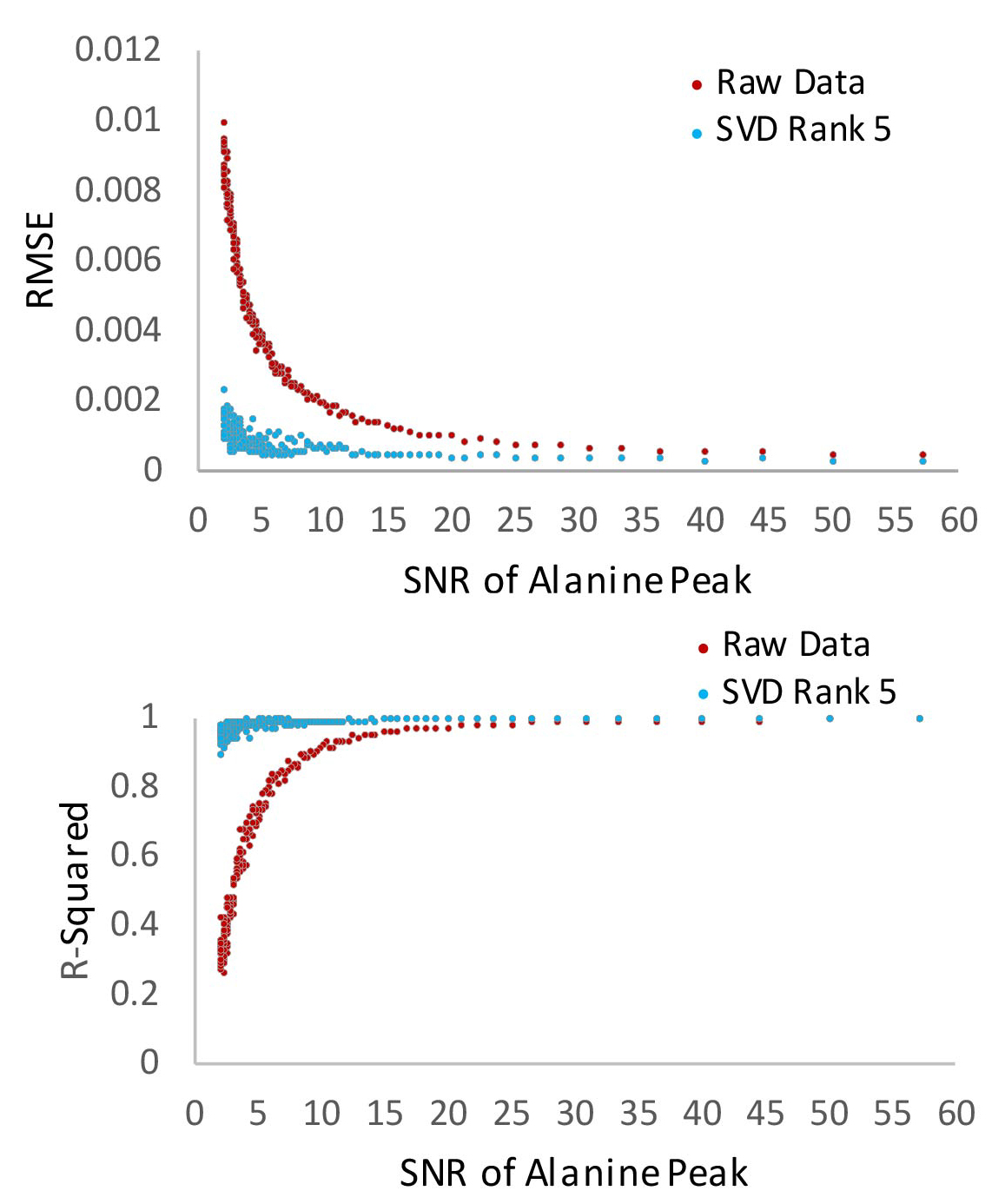


**Figure S2** Precision of curve-fitting of the alanine peak in a synthetic data set expressed as **(A)** R-Squared or **(B)** Root Mean Squared Error (RMSE). Use of rank reduction by SVD yielded precise curve-fitting over a large range of signal to noise, while the precision of curve-fitting of the raw data to the biexponential equation $y=A(e^{-k_{1}t}-e^{-k_{2}t}$) declined dramatically when the SNR of the metabolite peak became less than 10.


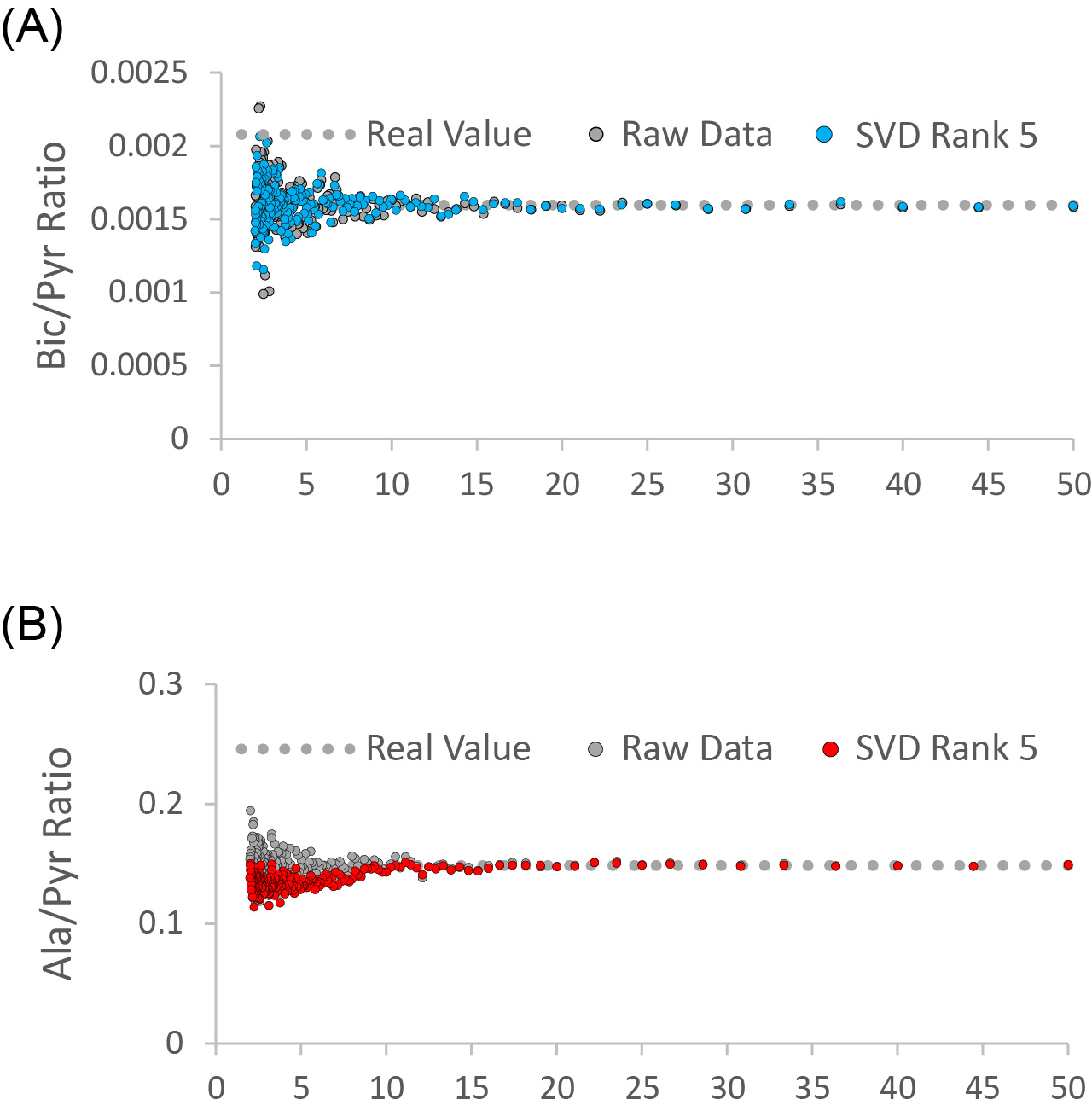


**Figure S3** A­­­­­­ccuracy of SVD rank reduction from reconstruction of a simulated data set. (A) Bicarbonate-to-pyruvate ratios as a function of the SNR of the bicarbonate peak from the raw and a rank 5 SVD reconstruction (B) Same as above except for the pyruvate peak. A slight bias in the SVD reconstruction exists for the alanine peak at low (<10) signal-to-noise ratios. SVD reconstruction yielded an unbiased estimate at all noise levels for the bicarbonate peak.

­­


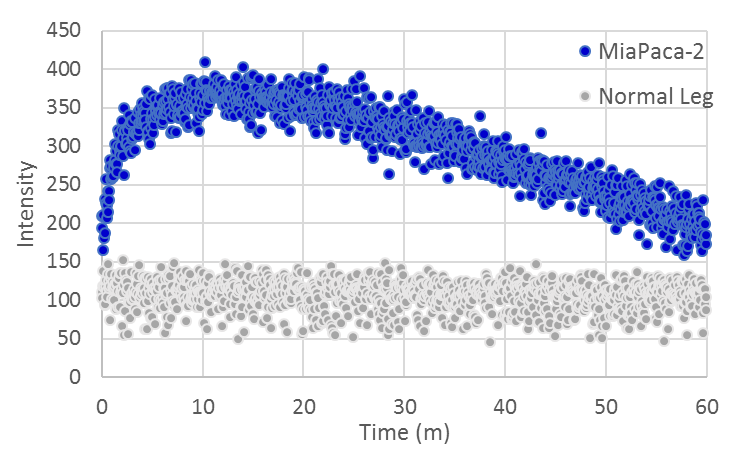

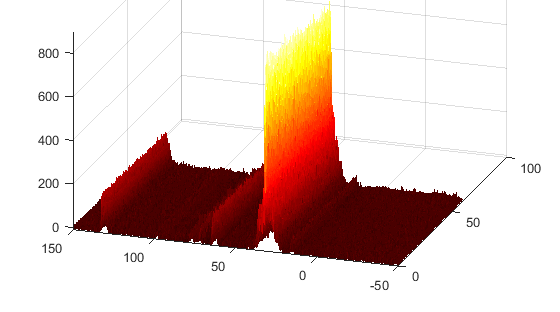

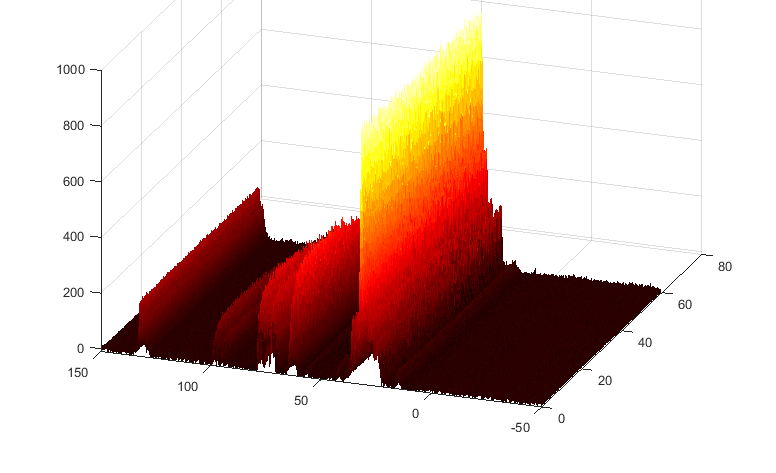

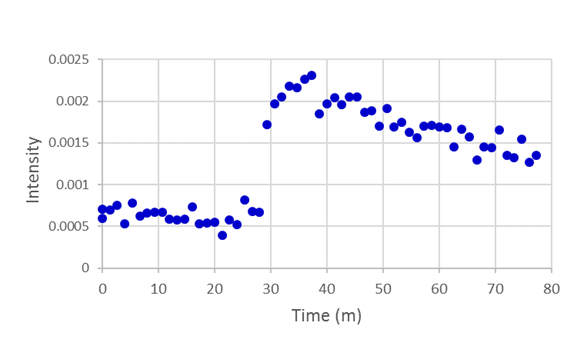

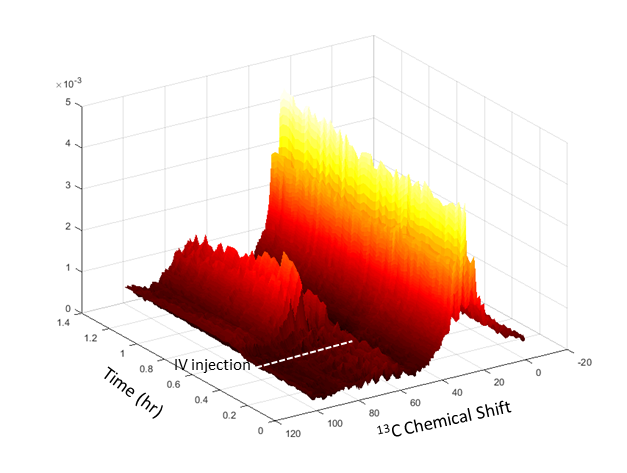


**Figure S4 (A)** Evolution of a hyperpolarized U-^13^C-^2^H glucose tracer after being injected directly into an Hs766t leg xenograft. **(B)** The spectrum at the point of maximum intensity at 24 seconds. Despite the high signal to noise, the only detectable peaks in the spectrum are from glucose with no evidence of metabolic turnover. **(C)** The same spectrum after rank reduction to a rank of 3. A doublet near the expected position of lactate is now apparent. (**D** and **E**). Kinetics of the main glucose and lactate peaks. The lactate peak decays more slowly than the pyruvate peak, reflecting the eventual breakdown of glucose into lactate.

**(A)**


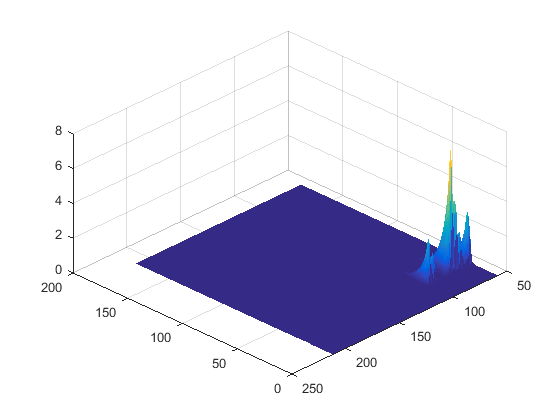


Time (s)

^13^C Chemical Shift


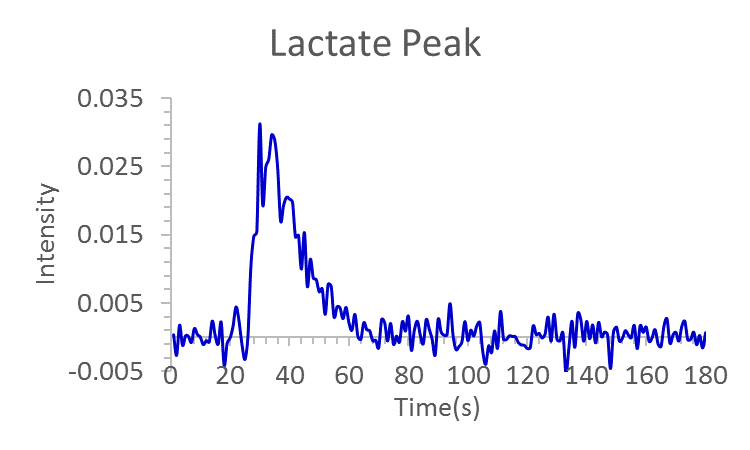

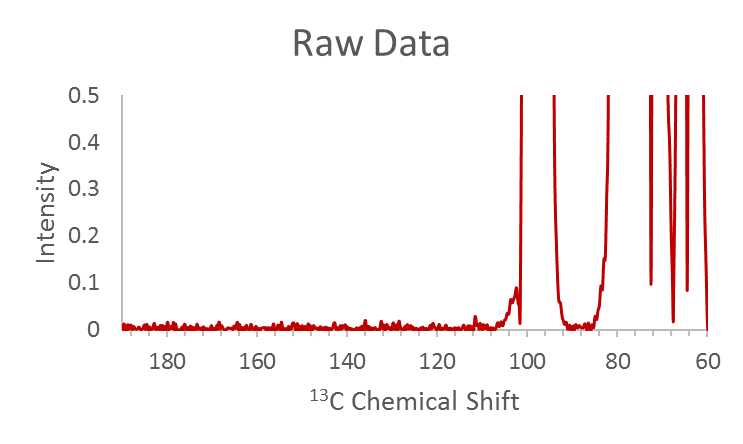

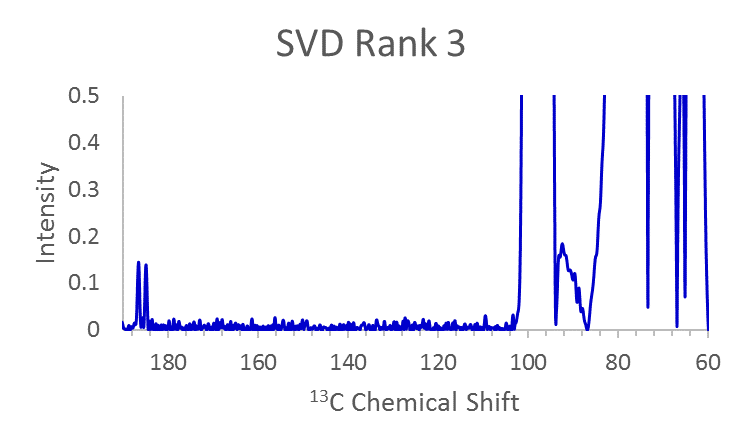

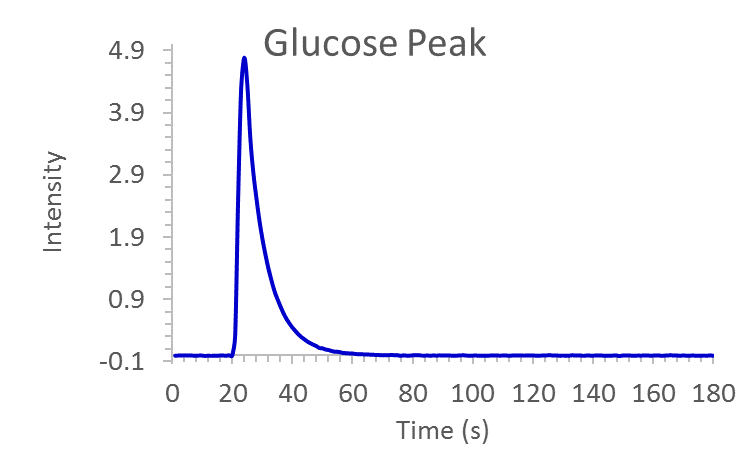


**(B)**

**(C)**

**(D)**

**(E)**

**Figure S5 Comparison of Glucose Metabolism** ^13^C signal after injecting 50 mg of uniformly ^13^C labeled glucose into the tail vein of a mouse with a MiaPaca xenograft without hyperpolarization after rank reduction. **(B)** The same experiment on a mouse without a tumor xenograft. **(E)** Kinetics of the signal at 60.5 ppm. No uptake is detectable in the leg without a xenograft, only the constant background signal from lipid glycerol groups is visible.

**(A)**

**(B)**

**(C)**

**Figure S6: (A)** Evolution of the ^13^C signal at 3T after IV injection of 50 mg U-^13^C glucose. The dashed line indicates the time of injection. **(B)** Kinetics of the glucose signal.

**(A)**

**(B)**


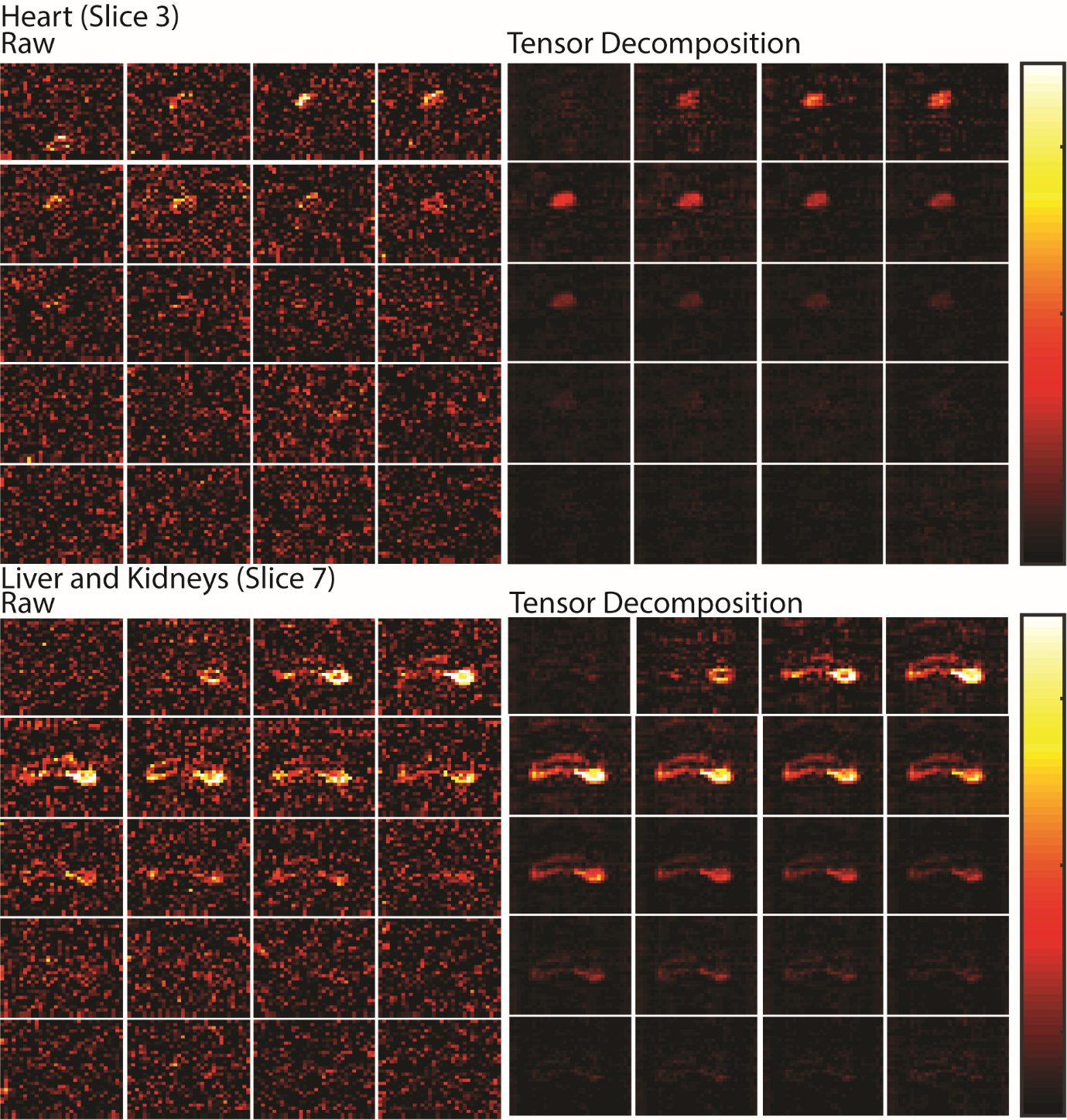

Supplement: Supplementary file 1 — Supplementary Information [file 41598_2019_38981_MOESM1_ESM.docx]
